# The influence of gender concordance between general practitioner and patient on antibiotic prescribing for sore throat symptoms: a retrospective study

**DOI:** 10.1186/s12875-018-0859-6

**Published:** 2018-11-17

**Authors:** D. Eggermont, M. A. M. Smit, G. A. Kwestroo, R. A. Verheij, K. Hek, A. E. Kunst

**Affiliations:** 1 Department of Public Health, Amsterdam UMC, University of Amsterdam, Meibergdreef 9, Amsterdam, 1105 AZ the Netherlands; 2 Netherlands Institute for Health Services Research (Nivel), Otterstraat 118-124, Utrecht, 3513 CR the Netherlands

**Keywords:** Gender role, Anti-bacterial agents, Drug resistance, Prescriptions, Sore throat, General practitioner

## Abstract

**Background:**

Patient gender as well as doctor gender are known to affect doctor-patient interaction during a medical consultation. It is however not known whether an interaction of gender influences antibiotic prescribing. This study examined GP’s prescribing behavior of antibiotics at the first presentation of patients with sore throat symptoms in primary care. We investigated whether GP gender, patient gender and gender concordance have an effect on the GP’s prescribing behavior of antibiotics in protocolled and non-protocolled diagnoses.

**Methods:**

We analyzed electronic health record data of 11,285 GP practice consultations in the Netherlands in 2013 extracted from the Nivel Primary Care Database. Our primary outcome was the prescription of antibiotics for throat symptoms. Sore throat symptoms were split up in ‘protocolled diagnoses’ and ‘non-protocolled diagnoses’. The association between gender concordance and antibiotic prescription was estimated with multilevel regression models that controlled for patient age and comorbidity.

**Results:**

Antibiotic prescription was found to be lower among female GPs (OR 0.88, CI 95% 0.67–1.09; *p* = .265) and female patients (OR 0.93, 95% 0.84–1.02; *p* = .142), but observed differences were not statistically significant. The difference in prescription rates by gender concordance were small and not statistically significant in non-protocolled consultations (OR 0.92, OR 95% CI: 0.83–1.01; *p* = .099), protocolled consultations (OR 1.00, OR 95% CI: 0.68–1.32; *p* = .996) and all GP practice consultations together (OR 0.92, OR 95% CI: 0.82–1.02; *p* = .118). Within the female GP group, however, gender concordance was associated with reduced prescribing of antibiotics (OR 0.85, OR 95% CI: 0.72–0.99; *p* = 0.034).

**Conclusions:**

In this study, female GPs prescribed antibiotics less often than male GPs, especially in consultation with female patients. This study shows that, in spite of clinical guidelines, gender interaction may influence the prescription of antibiotics with sore throat symptoms.

## Background

Bacterial resistance is an important topic in today’s health policy [1]. Although growing microbacterial resistance is partly a natural process, it can be accelerated with the inappropriate prescription of antibiotics by health care professionals [2]. With this in mind, factors which influence the prescription of antibiotics, other than patient’s clinical presentation, became an object of study. Indeed, research suggests the importance of such non-medical factors. General practitioners’ (GPs) attitudes such as fear and complacency affect prescribing behavior [3, 4]. Akkerman showed that physicians with more years of practice were more likely to prescribe antibiotics, especially if they felt they had little time per patient [5]. Moreover, GP’s perception of patients’ expectations concerning medication prescription influences the actual prescribing behavior [6–8]. Overall, these studies imply that doctor-patient interaction has an important influence on antibiotic prescribing behavior.

When focusing on the doctor-patient relationship, the gender of both the doctor and patient are known to influence their interaction [9, 10]. Among others, the GP’s gender may determine the communication style, the contents of the consultation, which drugs are indicated and whether they are prescribed [11–13]. In turn, the gender of the patient also affects the interaction between doctor and patient. For example, female patients are more likely to express their emotions during the consultation [14] and had fewer discussions about addictive behavior or heart disease risk [9, 15]. This implies that GPs might make medical decisions which are affected by gender-related considerations and gender stereotypes.

Since both patient’s and doctor’s gender play a part in the medical process, it is not unlikely that the combination of a patient and doctor of the same gender (compared to dyads of opposite gender) may have additional effects. In interactions between patient and doctor, four gender dyads can be distinguished (male-male, female-female, male-female, female-male) of which two are concordant and two are discordant. It has been found that female-female consultations contain more affective talk and less analytical talk. The opposite occurs in male-male consultations [16]. Moreover, it appears that female concordance leads to communication that is most patient centered [17, 18], which may enhance health outcomes by elevated patients’ trust, improved communication and patient satisfaction. Indeed, female gender concordance is associated with more effective treatment of cardiovascular risks [19] and male gender concordance is positively associated with measures on diet, nutrition and exercise counseling [20]. It is however not known whether gender concordance influences prescribing behavior of antibiotics. Possibly, concordance is an additional non-medical factor that affects (inappropriate) antibiotics prescription. Creating awareness of such non-medial factors could result in GPs being less biased, more objective and consistent in their treatments.

Since the 1980’s there is an increase in the extent to which primary care practice is influenced by clinical guidelines [21]. For example, some diagnoses are followed by guidelines that leave the physician room to follow a treatment of choice (‘non-protocolled’ guidelines). Other diagnoses have guidelines in which a ‘protocolled’ treatment (e.g. antibiotic prescription) is strongly recommended. The power of a clinical guideline is to make sure the patient gets the most effective, evidence-based treatment. A guideline also reduces the variation in treatment for the same diagnoses between GPs.

In this study, we will explore whether the prescription of antibiotics depends on the patients or physicians gender and/or on their gender interaction (concordance). To do this, we will study prescribing behavior concerning patients presenting sore throat symptoms in primary care. This patient group was chosen since the inappropriate prescription of antibiotics is very common in sore throat symptoms [22] and because the majority of antibiotic prescriptions in The Netherlands is issued in primary care [23]. In addition, we will investigate whether gender concordance is less influential in diagnoses corresponding to a strong antibacterial protocol than in diagnoses that leave the GP more room for interpretation and choice.

## Methods

The specific objectives of this study were to explore:

1. whether the likelihood that patients will be given a medical prescription depends on the gender of, respectively, the patient and the GP.
2. whether this likelihood depends on the gender concordance between patient and GP, with distinction between male-male and female-female concordance.
3. the role of gender and of gender concordance for prescription policy in non-protocolled guidelines and protocolled guidelines.

### Data source

The electronic health records for this study were provided by the Netherlands Institute for Health Services Research (Nivel) Primary Care Database, containing GP practice consultations from 2013. We included general practices that registered information on the function and sex of caregiver. Moreover, the data included information on consultations, prescriptions (coded according to the Anatomical Therapeutical Chemical (ATC) classification) and diagnosis (coded according to the International Classification of Primary Care (ICPC) version 1) [24]. These consultations were handled by health professionals of whom 225, according to information provided by the practice, were known to be trained as GP. Other health professionals were for example physiotherapist, physician assistant, dietician or practice nurse. We used data from 22,412 GP practice consultations concerning patients with sore throat symptoms.

### Variables

The electronic health records data contained information on: prescription of antibiotics (did or did not prescribe a medicine with ATC-code J01, which are antibiotics for systemic use), sex of both patient and caregiver, age of patient, function of caregiver (e.g. physiotherapist, physician assistant, dietician, nurse etc.), comorbidity (did or did not suffer from one or more chronic disease according to the GP’s medical file) and ICPC-code. The ICPC classification system is used to record diagnosis and/or symptoms. This study focuses on ICPC codes relating to sore throat symptoms (see Table 1). Using the ICPC code, we distinguished between diagnoses based on symptoms versus those based on underlying pathology. ICPC-codes R21 and R22 represent symptoms (e.g. coughing) indicating that at the time of the consultation no real diagnosis (e.g. tonsillitis) was apparent.

**Table 1:** Diagnoses for sore throat symptoms

ICPC-code	Diagnoses	Percentage of all consultations (*n* = 11,285)	Protocolled	Symptoms/no diagnosis
R21	Symptoms/complaints throat	19.8%	No	Yes
R22	Symptoms/complaints tonsils	1.0%	No	Yes
R72	Streptococcus/scarlet fever	1.3%	No	No
R74	Acute infection upper airway	67.4%	No	No
R76	Acute tonsillitis/peritonsillar abscess	8.5%	Yes	No
R77	Acute laryngitis/tracheitis	1.9%	No	No

We also measured whether the disease was ‘protocolled’. For this, the ICPC code R76 (acute tonsillitis/peritonsillar abscess) was regarded as protocolled. The Dutch GP guideline for acute sore throat complaints firmly advises to prescribe antibiotics for this diagnosis [25]. Other ICPC-codes related to sore throat were classified as non-protocolled, i.e. antibiotics are not indicated. To determine the presence of an antibiotic treatment protocol, the GP guideline was analyzed independently by three coders. Intercoder agreement was 100%, as all coders regarded the same ICPC-codes as either ‘protocolled’ and ‘non-protocolled’.

### Exclusion criteria

The following exclusion criteria were used to select patients and GP practice consultations for the analyses:

1. This study focuses on the gender interaction between patient and GP in the general practice. Hence, all practice consultations with other health care professionals within the practice (e.g. medical student, physician assistant, dietician, etc.) were excluded.
2. In the communication between a GP and a child, parents are usually involved [26]. Consequently, the gender interaction between child and GP is complicated by the gender of the parent that is present during the consultation. Therefore, all children (age 0 to 17) were excluded from the study.
3. At first presentation, all treatment options are still possible and patient-doctor interaction may be decisive for prescription. Therefore, we decided to focus on first consultations, which implied that all second and consecutive practice consultations in 2013 for sore throat symptoms were excluded.

### Descriptives and demographics

**Fig. 1:**
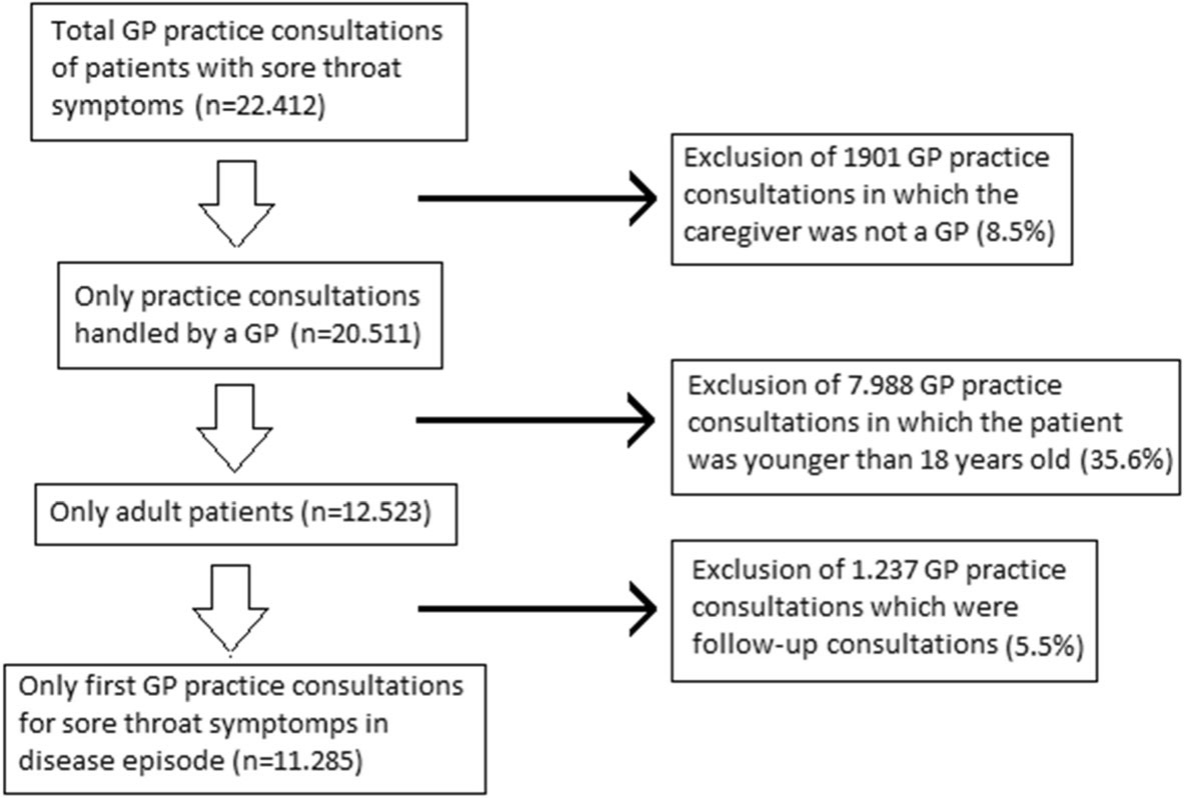
Exclusion process

After selecting only the practice consultations handled by a GP, 20511 (91.5%) consultations were available for analysis (see Fig. 1). The second exclusion criterion (only 18+ patients) brought our dataset back from 20,511 to 12,523 (61.1%) consultations. Finally, after applying our last exclusion criterion (only first presentation), 11,285 (55.0%) first consultations, handled by 225 GPs, remained for analysis.

### Statistical analyses

Logistic regression was performed to calculate the effect of our main predictors on our outcome variable ‘prescription of antibiotics’ (dichotomous). In the regression model, our main predictors for antibiotic prescription were patient gender, GP gender and concordance. Patient’s age and comorbidity were added as control variables. We also tested whether GP’s gender and gender concordance had an interaction effect by creating an interaction term and adding this to the regression model along with its constituting variables. In addition, in further analyses, the regression models were calculated separately for protocolled and non-protocolled prescriptions. In order to explore whether the main predictors have different effects on male and female GP’s, the regression models were also calculated separately for male and female GP’s consultations.

Due to clustering of observations at the level of GPs and health practices, we applied multi-level models. In a generalized linear mixed model, we entered three different data levels: [1] practice, [2] GP and [3] patient. All analyses were performed with statistical package SPSS versions 22.

### Privacy

Dutch law allows the use of electronic health records for research purposes under certain conditions. According to the legislation, neither obtaining informed consent from patients nor approval by a medical ethics committee is obligatory for this type of observational studies containing no directly identifiable data [27]. This study has been approved by the applicable governance bodies of NIVEL Primary Care Database under number NZR-00315.025.

## Results

Of all GP practice consultations, 52.1% were handled by male GPs. In 39.2% of the consultations the patient was male. Of all cases 27.6% of the patients were prescribed antibiotics. The concordant dyads are represented with 52.8% of the consultations, while 47.2% of the consultations contained discordant couples. For all consultations, 8.5% got assigned an ICPC code labeled as protocolled and 91.5% as non-protocolled.

Table 2 shows that patient age, presence of comorbidity and the amount of consultations without a real diagnosis were similar in consultations with male and female GPs. Male GPs prescribed antibiotics in 29.5% of their consultations. Female GPs did so in 25.7% of their consultations. For female GPs, 64.1% of their consultations were concordant (i.e. with a female patients), while male GPs had same-sex consultations in 42.3% of their consultations. The concordant couples had an antibiotic prescription rate of 26.8% of the consultations, against 28.6% in discordant couples.

**Table 2:** Descriptive of patients seen by male GP vs female GP and concordant vs discordant couples

	Male GP	Female GP	Concordant	Discordant
Sample size	5956	5329	5956	5329
Average age patient (years)	46.7	46.3	46.6	46.4
Comorbidity patient	53.6%	52.5%	53.3%	52.7%
Concordance	42.3%	64.1%	–	–
Symptoms / no diagnosis	20.3%	21.4%	21.5%	20.1%
Protocolled diagnoses	7.9%	9.1%	8.4%	8.6%
Antibiotics prescribed				
All diagnoses	29.5%	25.7%	26.8%	28.6%
-Male GP	–	–	29.6%	29.4%
-Female GP	–	–	24.8%	27.3%
In non-protocolled diagnoses	25.6%	21.5%	22.8%	24.6%
-Male GP	–	–	25.8%	25.5%
-Female GP	–	–	20.7%	23.0%
In protocolled diagnoses	74.1%	67.3%	69.7%	71.7%
-Male GP	–	–	76.9%	72.3%
-Female GP	–	–	65.5%	70.7%

**Fig. 2:**
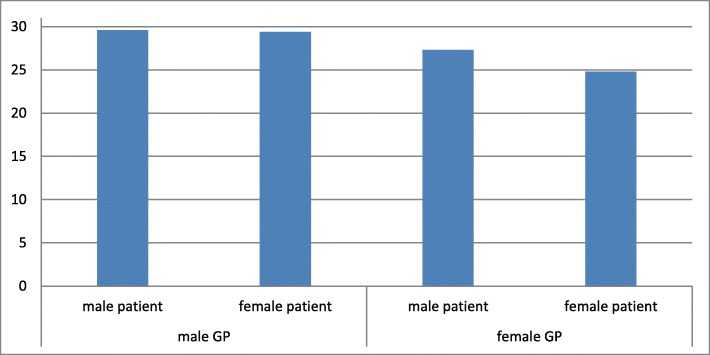
Percentage of consultations for sore throat symptoms with antibiotic prescription. Female GP’s prescribe antibiotics less often than male GP’s (*p* = .000). In dyads with a female GP, antibiotics are less often prescribed when there is gender concordance (*p* = .044)

Figure 2 shows the percentage of antibiotics prescribed per gender dyad. In accordance with Table 2, it illustrates that female GPs prescribe less antibiotics than male GPs. Female GPs especially prescribe less antibiotics for female patients (24.8% in concordant couples versus 27.3% in discordant couples). In consultations with male GPs, on the other hand, there is no such difference (concordant couples 29.6% versus 29.4% in discordant couples).

Table 3 shows the outcome of the multilevel regression analysis. In the total population, concordance (OR 0.92, OR 95% CI: 0.83–1.01), female GP (OR 0.88, 95% Cl: 0.67–1.09) and female patient (OR 0.93, 95% Cl: 0.84–1.02) show lower rates of antibiotic prescription, however not with statistical significance. Similar results were found in both types of prescriptions (protocolled and non-protocolled). Patient age was significantly associated with antibiotics prescription (OR 1.00, 95% CI: 1.00–1.00). This was equally true for protocolled and non-protocolled consultations. Comorbidity was a significant predictor in non-protocolled consultations (OR 1.21, 95% CI: 1.01–1.32), but not in the protocolled consultations (OR 0.92, 95% CI: 0.57–1.26).

**Table 3:** Predictors for antibiotic prescription after multi-level analysis^a^

	Exp (B)	Lower	Upper	Significance
All GP practice consultations (*n* = 11,285)^b^				
Concordance	0.92	0.83	1.01	*p* = .099
Gender GP	0.88	0.67	1.09	*p* = .265
Gender patient	0.93	0.84	1.02	p = .142
Comorbidity	1.09	0.99	1.18	*p* = .090
Age patient	1.00	1.00	1.00	*p* = .022
Non-protocolled (*n* = 10,328)^c^				
Concordance	0.92	0.82	1.02	*p* = .118
Gender GP	0.83	0.58	1.08	*p* = .180
Gender patient	0.96	0.85	1.06	*p* = .404
Comorbidity	1.21	1.01	1.32	*p* = .000
Age patient	1.00	0.99	1.00	*p* = .000
Protocolled (*n* = 957)^d^				
Concordance	1.00	0.68	1.32	*p* = .996
Gender GP	0.65	0.26	1.04	*p* = .076
Gender patient	0.79	0.47	1.10	*p* = .184
Comorbidity	0.92	0.57	1.26	*p* = .633
Age patient	0.98	0.96	0.99	*p* = .002

^a^These findings are based on mulit-level logistic regression analysis

^b^There was no interaction effect of concordance and gender GP (*p* = .225)

^c^There was no interaction effect of concordance and gender GP (*p* = .272)

^d^There was no interaction effect of concordance and gender GP (*p* = .145)

Table 4 shows that for female GPs concordance is associated with antibiotic prescription after controlling for comorbidity and patient age. In female concordant consultations less antibiotics were prescribed (OR 0.85, 95% CL: 0.72–0.99). The lower section of Table 4 shows that for male GPs concordance is not associated with antibiotic prescription after controlling for comorbidity and patient age (OR 0.99, 95% CL: 0.86–1.11).

**Table 4:** The model of predictors for antibiotic prescription according to gender of GP^a^

	Practice consultations with female GP (*n* = 5410)				Practice consultations with male GP (*n* = 5875)			
	Exp (B)	Lower	Upper	Significance	Exp (B)	Lower	Upper	Significance
Concordance	0.85	0.72	0.99	*p* = .034	0.99	0.86	1.11	*p* = .827
Comorbidity	1.16	1.02	1.31	*p* = .028	1.02	0.89	1.16	*p* = .731
Age patient	1.00	1.00	1.01	*p* = .063	1.00	1.00	1.00	*p* = .193

^a^These findings are based on mulit-level logistic regression analysis

## Discussion

### Summary of results

In this study, we assessed whether gender concordance between GP and patient was associated with antibiotics prescribing. Gender concordance among females is associated with less prescription of antibiotics, i.e. female GPs appear to issue fewer antibiotics prescriptions to female patients than to male patients. Concordance appears not to have a similar effect in prescribing behavior of male GPs. For both concordant dyads combined, prescription rates were lower than for discordant dyads, but this difference was not statistically significant. Whether the treatment was protocolled did not appear to affect these findings.

### Interpretation of results

When a patient with sore throat symptoms presents him- or herself in a primary care facility, infection is usually the underlying cause [28]. For these symptoms, physicians can send their patient home for a ‘wait and see’ policy or they can prescribe antibiotics. The results of this study indicate that female physicians are more likely to apply a wait and see policy when seeing a female patient in comparison with the other gender dyads.

Female concordance has been associated with a communication style that is more patient centered [17, 18]. Patient centered communication enhances health outcomes by elevated patients’ trust, improved communication and patient satisfaction [17–20, 29]. When communication is patient centered, the physician focuses at hearing and understanding the patients’ perspectives. For this, the physician needs to explore the patients ideas and concerns as well as their expectations regarding the physician [30]. It has been shown that addressing patients’ ideas, concerns and expectations during a consultation, known as the ICE-model, might lead to fewer medication prescriptions [31]. This is potentially important because a wait and see policy is easier to adopt when patients feel being heard.

In our study, 27.6% of all patients got prescribed antibiotics for their sore throat complaints. This is low in comparison to other countries. In the UK, for example, approximately 56% of all patients with sore throat complaints receive antibiotics from their GP [32]. Traditionally, antibiotic prescription in the Netherlands is lowest of all European countries, with differences up to a threefold [33]. It is unclear how this relates to the outcomes of our study. Therefore, generalizing our findings to countries with much higher antibiotic prescription rate should be done with caution.

Patient age and comorbidity were identified as being predictive for prescribing antibiotics. This was in line with previous research which showed age to be a determinant in the prescription of antibiotics for lower respiratory tract infections and that antibiotics are more often prescribed for patients with comorbidity such as diabetes or chronic obstructive pulmonary disease [34].

### Limitations of the study

This study was performed with electronic health records data that were routinely recorded in general practices. This type of data has advantages and disadvantages. Advantages are that the data is cheap, readily available and can be assumed in many respects to represent what actually takes place in clinical practice. However, it also means that circumstances, habits and customs of individual practices in the way data are recorded, can have an impact on the quality of the data [35]. In this study this may have contributed to the fact that we had to exclude 8.5% of all consultations. These consultations had to be excluded because these consultations did not appear to have taken place with the GP but with other practice personnel. The percentage of consultations with the GP is likely to be an underestimate. Some of the consultations may have been taken care of by the GP, but recorded by a practice assistant.

In this study, we would have liked to include age of the GP. It has been shown that the years of experience of the GP correlates with antibiotics prescription [5]. Because, on average, male GPs are older than female GPs [36], age might have been a confounder in this study. Another limitation is that we could not include information on patients’ expectations and concerns, which might also influence prescribing behavior [31].

Although the existence of comorbidity was reported by the GP, in this study we regarded comorbidities as present or non-present and did not investigate the role of individual comorbidities. However, COPD and diabetes are examples of diseases which affect susceptibility to disease and therefore possibly influence the prescribing behavior of the GP to a greater extent than most other diseases. In future research it would be important to include these comorbidities separately.

The current study focused solely on sore throat. Therefore, generalizing our findings to other complaints or symptoms should be done with caution. Future research should assess for a broader spectrum of symptoms whether prescription rates are related to gender concordance. Also, sore throat symptoms can be considered relatively gender neutral. This would be different when for example urogenital symptoms were concerned. This could change the effect of gender concordance. Further research could focus on the effect of gender concordance on antibiotics.

### Implications of this study

In recent years the medical community in western societies has experienced a growth in number of female physicians [36]. The results of this study suggest that this feminization could lead to a reduction in the prescription of antibiotics. Female concordance enhances patient centred communication and this might be the underlying explanation for our findings. If so, our results underline the importance of effective communication styles, both by male and female GPs, to contain the prescription of antibiotics.

## Conclusion

In this study, we found that gender concordance among females is associated with less prescription of antibiotics for sore throat complaints. In other words, female GPs appear to issue fewer antibiotics prescriptions to female patients than to male patients. Among male GPs, concordance did not appear to have a similar effect. Possibly, our findings can be explained by a different communication style that is handled by female GPs, especially when attending with female patients. Future studies should aim to describe and understand female concordance in more detail, since the findings may suggest that training doctors to adjust their communication style could prevent the prescription of unnecessary antibiotics.

## Abbreviations

ATC: Anatomical therapeutical chemical
GP: General Practitioner
ICPC: International classification of primary care
Nivel: Netherlands institute for health services research
